# Hyaluronate Functionalized Multi-Wall Carbon Nanotubes Loaded with Carboplatin Enhance Cytotoxicity on Human Cancer Cell Lines

**DOI:** 10.3390/ma14133622

**Published:** 2021-06-29

**Authors:** César Adrián Leyva-González, Daniel Salas-Treviño, Flavio Fernando Contreras-Torres, María de Jesús Loera-Arias, Christian Alexis Gómez-Tristán, Edgar Iván Piña-Mendoza, Gerardo de Jesús García-Rivas, Gloria Arely Guillén-Meléndez, Roberto Montes-de-Oca-Luna, Odila Saucedo-Cárdenas, Adolfo Soto-Domínguez

**Affiliations:** 1 Departamento de Histología, Facultad de Medicina, Universidad Autónoma de Nuevo León, Monterrey C.P. 64460, Mexico; cesar.leyvagn@uanl.edu.mx (C.A.L.-G.); daniel.salastr@uanl.edu.mx (D.S.-T.);mdjesus.loeraars@uanl.edu.mx (M.d.J.L.-A.); christian.gomeztrs@uanl.edu.mx (C.A.G.-T.); edgar.piname@uanl.edu.mx (E.I.P.-M.); gloria.guillenmln@uanl.edu.mx (G.A.G.-M.); robertomontesdeocaln@uanl.edu.mx (R.M.-d.-O.-L.); 2 Escuela de Ingeniería y Ciencias, Tecnológico de Monterrey, Monterrey C.P. 64849, Mexico; contreras.flavio@tec.mx; 3 Tecnológico de Monterrey, Escuela de Medicina, Monterrey C.P. 64849, Mexico; gdejesus@tec.mx; 4 Departamento de Genética Molecular, Centro de Investigación Biomédica del Noreste (CIBIN) del IMSS, Monterrey C.P. 64720, Mexico

**Keywords:** cytotoxicity, human cancer cells, multiple-walled carbon nanotubes, hyaluronic acid, carboplatin

## Abstract

Cancer is a major global public health problem and conventional chemotherapy has several adverse effects and deficiencies. As a valuable option for chemotherapy, nanomedicine requires novel agents to increase the effects of antineoplastic drugs in multiple cancer models. Since its discovery, carbon nanotubes (CNTs) are intensively investigated for their use as carriers in drug delivery applications. This study shows the development of a nanovector generated with commercial carbon nanotubes (cCNTs) that were oxidized (oxCNTs) and chemically functionalized with hyaluronic acid (HA) and loaded with carboplatin (CPT). The nanovector, oxCNTs–HA–CPT, was used as a treatment against HeLa and MDA–MB-231 human tumor cell lines. The potential antineoplastic impact of the fabricated nanovector was evaluated in human cervical adenocarcinoma (HeLa) and mammary adenocarcinoma (MDA-MB-231). The oxCNTs–HA–CPT nanovector demonstrate to have a specific antitumor effect in vitro. The functionalization with HA allows that nanovector bio–directed towards tumor cells, while the toxicity effect is attributed mainly to CPT in a dose-dependent manner.

## 1. Introduction

Cancer is defined as the set of diseases in which abnormal cells grow, multiply uncontrollably, and can invade adjacent tissues or spread through the blood or lymphatic system and metastasize [1]. Cancer is the second cause of death globally, producing 1 of 6 deaths, and this condition represents a significant public health problem [2]. Current cancer therapies include surgery, radiotherapy, immunotherapy, targeted therapy, hormonal therapy, and/or chemotherapy [3], and their use depends on the nature, location, and stage of the tumor. Chemotherapy is the primary treatment of choice in most advanced-stage cancers [3].

Carboplatin (CPT) is a chemotherapeutic drug widely used for various types of cancer; however, its use is restricted due to the severe adverse effects. To avoid these effects, it is necessary to reduce the dose of the drug, which may reduce the effectiveness to treat the original problem [4]. However, under several circumstances, the adverse effects cause considerable complications, including insensitivity to anticancer therapy and acquiring the so-called chemoresistance [5].

Currently, modern technologies have been developed to optimize the delivery of antineoplastic drugs. In this area, the development of drug delivery systems has played an essential role especially in those therapies directed against cancer. The physicochemical properties of nanomaterials, the versatility to be functionalized, and the possibility to bio-direct them towards tumor cells using ligand receptors are intensively applied for biomedical purposes related to targeted delivery treatments, pharmacological or gene therapy, and cell differentiation [6,7]. For instance, nanocarriers loaded with antineoplastic drugs have demonstrated better effectiveness against chemoresistant neoplasms [6].

Carbon nanotubes (CNTs) are the referent nanomaterial for novel applications in nanomedicine [8]. CNTs consist of rolled cylindrical sheets of graphene with sp2-hybridizing carbon atoms. Multi-walled CNTs (MWCNTs) consist of two or more graphene sheets wound on themselves whose diameter is between 2–50 nm depending on the number of graphene layers that compose them [9]. CNTs are insoluble in water due to their chemical composition, which represents a problem for their application as drug-releasing nanosystems. To overcome this, their surface must be modified through a process known as derivatization (i.e., chemical functionalization) [10,11]., which can bind hydrophilic molecules via covalent bonds. The chemical modifications onto the surface of CNTs can facilitate a further interaction with other molecules, not only to increase dispersion in aqueous media [12], but also to incorporate specific ligands used in biological systems [13]. Primary amines (NH2), folic acid (FA), and hyaluronic acid (HA), polyethylene glycol (PEG), and epidermal growth factor (EGF) are often used to functionalize CNTs [12]. The wide variety of functional groups attached to the walls of CNTs enables the bonding of ligand molecules that yield specificity to the nanovector, such as HA and its receptor CD44 because this binding stimulates receptor-mediated endocytosis [13]. The CD44 receptor is a transmembrane glycoprotein that is overexpressed in tumor cells related to the aggressive stages of various types of cancer [14]. The activation of this receptor induces the proliferation, adhesion, migration, and survival of tumor cells [15].

With the increased use of commercial CNTs (cCNTs) in different therapeutic settings, the potential adverse effects of those nanomaterials require to be carefully evaluated, especially in anticancer therapies [16]. For instance, pristine (i.e., unfunctionalized) CNTs are known to form aggregates that cause fibrosis, granulomatous lesions, oxidative stress among other toxic effects [17,18,19,20,21,22]. MWCNTs can be readily taken up by HeLa cells [23] with significantly different cell uptake efficiencies with graphene oxide. Although the cell uptake ratio of MWCNTs was higher than that of Graphene Oxide (GO), their cytotoxicity showed no significant difference. However, the potential release of pristine CNTs in biodegradation and the possible related toxicity is still an insistent question with controversial replies [24]. Several studies have been performed in the last years on the toxicological effects of CNTs. However, existing data are controversial: fiber length and diameter, surface area, a tendency to agglomerate, dispersibility in media, impurities, and presence of metal catalyst due to the method of production influence toxicity and reactivity of CNTs [25].

Recent studies showed the development of HA-modified MWCNTs as an efficient anticancer drug nanocarrier for tumor-targeted chemotherapy when loaded with doxorubicin [26]. Exposure to the NH2-functionalized carbon nanotubes loaded with CPT resulted in a more significant decrease in viability than oxidized CNTs (oxCNTs) loaded with the same drug [27]. CPT-loaded PEGylated-modified MWCNTs, however, turned out to be ineffective on the viability of MDA-MB-231 (human breast cancer) cells [28]. Previously, in our research team, we described a new antineoplastic therapeutic strategy using a nanovector fabricated with MWCNTs and functionalized with HA to be loaded with CPT [29]. Such MWCNTs–HA–CPT nanovector showed selective cytotoxic activity against TC-1 murine lung cancer tumor cells. The cytotoxic effect of the constructed nanovector was shown to be at least twice as high compared to CPT. Even though the TC-1 cell line has good characteristics to test our nanovector in the context of a tumor model, these cells are mouse pulmonary epithelial cells transformed with HPV-16 E6 and E7 proteins and form metastatic tumors, specifically in the lungs when inoculated intravenously. Therefore, the primary objective of the present study was to analyze in vitro cytotoxic activity for the prepared nanovectors against human cancer cells. This study compares a different behavior and sensitivity between human cervical adenocarcinoma (HeLa) and mammary adenocarcinoma (MDA-MB-231) when treated with the cCNTs, oxCNTs, oxCNTs–HA, oxCNTs–HA–CPT, and CPT. Embryonic mouse fibroblast (NIH-3T3), a suitable transfection host non-tumor cell line, was used to compare the susceptibility to treatments with nanovectors.

## 2. Materials and Methods

### 2.1. Synthesis of the Graphical Nanovector Functionalized with HA and Loaded with CPT

The commercial-grade carbon nanotubes (cCNTs) were purchased from Nanostructured & Amorphous Materials, Inc. (NanoAmor^®^, Houston, TX, USA, Stock # 1245YJF, CAS # 99685-96-8). For the synthesis and characterization of the nanovector, the previously described methodology was used [29]. Briefly, oxidation of the cCNTs was performed using 50 mg of cCNTs, which were reacted with H_2_SO_4_ and HNO_3_ (3:1) in an ultrasound bath for 1 h and subsequently under magnetic stirring for 24 h. Oxidized CNTs (oxCNTs) were obtained, which were washed with distilled water until their pH was neutralized. Subsequently, the oxCNTs were dried in the oven at 60 °C for 48 h.

For the functionalization process, oxCNTs (30 mg) were dispersed into 12.5 mL of dimethylsulfoxide (DMSO) using an ultrasound bath for 30 min. On the other hand, an activating solution was prepared with 30 mg of N-ethyl-N’-(3-dimethylaminopropyl) carbodiimide hydrochloride (EDC-HCl, Sigma-Aldrich, #25952-53-8. St. Louis, MO, USA) and 15 mg of N-hydroxysuccinimide (NHS, Sigma-Aldrich, #6066-82-6. St. Louis, MO, USA) in 1.25 mL of DMSO, that was left stirring magnetically for 30 min. Both solutions were mixed and left under magnetic stirring for 3 h. After this reaction time, 30 mg of polyethyleneimine (PEI, Sigma-Aldrich, #9002-98-6. St. Louis, MO, USA) were added to the solution containing the oxCNTs allowing it to react for 48 h under magnetic stirring. With these reactions, PEI-functionalized oxCNTs (oxCNTs-PEI) were obtained. Then, the oxCNTs-PEI were washed with distilled water and they were left to dry in an oven at 60 °C for 48 h. Once recovered, 20 mg of the oxCNTs-PEI were taken and dispersed in 8 mL of DMSO on an ultrasound bath for 30 min. Next, an activating solution was prepared with 20 mg of EDC-HCl and 10 mg of NHS in 0.8 mL of DMSO. The activating solution was added to the oxCNTs–PEI solution and was left to react for 3 h under magnetic stirring. Subsequently, a solution of 20 mg of HA (Sigma-Aldrich, #9067-32-7. St. Louis, MO, USA) in 1.5 mL of DMSO was activated with 20 mg of EDC-HCl in 0.8 mL of DMSO. Then, the activated solution of HA was added to the solution with oxCNTs-PEI allowing it to react for 48 h in magnetic stirring. Finally, the oxCNTs–HA were washed with distilled water and left to dry for 48 h at 60°C for their subsequent recovery.

The process of loading the CPT in oxCNTs–HA was performed with the nanoextraction method. For this, 5 mg of oxCNTs–HA and CPT (Sigma-Aldrich, #41575-94-4. St. Louis, MO, USA) (5 mg/5 mL of pure ethanol) were mixed by magnetic stirring for 24 h. Subsequently, washes were performed with methanol, and the nanotubes were left to dry for 48 h for subsequent recovery, thus obtaining functionalized nanotubes loaded with CPT (oxCNTs–HA–CPT).

### 2.2. Characterization of cCNT and oxCNT-HA-CPT

The structure and morphology of both cCNT and oxCNT–HA–CPT were characterized using Transmission Electron Microscopy (TEM) with a Zeiss EM-109 microscope (Carl Zeiss AG, Jena, Germany) at 80 kV. The samples were prepared by dispersing the nanotubes in DMSO in an ultrasound bath for 30 min and deposited on lacey carbon grids with contrast 1% gold chloride. Micrographs of the nanotubes were obtained at 20,000×. Chemical characterization of the as-prepared nanovectors was recently reported in another study [29].

### 2.3. Establishment of Cell Lines

Three cell lines were used to analyze the in vitro cytotoxic effect of the oxCNT–HA and oxCNT–HA–CPT nanovectors. All cell lines were purchased from the American Type Culture Collection (ATCC) (Manassas, VA, USA).

(a) HeLa cell line: Cells derived from adenocarcinoma of the human cervix from a 31-year-old black female patient. They are adherent cells, of epithelial morphology and positive for keratin by immunoperoxidase staining. They have been reported to contain human papillomavirus type 18 sequences, as well as express low levels of p53 and normal levels of pRB. The cells were incubated in 1× Advanced DMEM medium supplemented with 4% v/v fetal bovine serum, 1% penicillin/streptomycin at 37 °C in a 5% CO_2_ atmosphere (ATCC^®^, CCL-2 ™) [30].
(b) MDA-MB-231 cell line: Cells derived from human mammary adenocarcinoma from a 51-year-old Caucasian female patient. They are adherent cells, of epithelial morphology and express the WNT7B oncogene. They express EGF and transforming growth factor-alpha (TGF-α). Cells were incubated in 1× Advanced DMEM medium supplemented with 4% v / v fetal bovine serum, 1% v / v penicillin/streptomycin at 37 °C in an atmosphere with 5% CO_2_ (ATCC^®^, HTB-26 ™) [31].
(c) NIH-3T3 cell line: Mouse embryonic fibroblasts. They are adherent cells with a tapered morphology. Suitable for transfections, however, in this study they were used to demonstrate susceptibility to oxCNT-HA-CPT. The cells were incubated in 1× Advanced DMEM medium, supplemented with 4% v/v fetal bovine serum, 1% v/v penicillin/streptomycin at 37 °C in an atmosphere with 5% CO_2_ (ATCC^®^, CRL-1658 ™) [32].

### 2.4. Immunofluorescence for the Determination of CD44 Receptor

The immortalized non-tumorigenic NIH-3T3 and the tumor cell lines HeLa and MDA-MB-231 were used. An indirect immunofluorescence technique was performed on them to determine the presence of the CD44 receptor. For this, 5 × 10^4^ cells were cultured in 24-well plates containing a round coverslip at the bottom of each well, the cells were left in incubation for 24 h. Subsequently, the cells were fixed with methanol for 20 min at 4 °C. The medium was removed from each well, washes were performed with phosphate buffer saline (PBS 1×), and then two washes with Tris and Tween 20 buffered saline (TBST). Once the washes had been performed, antigens were blocked with 10% horse serum for 1 h at room temperature (RT). Then, the primary rabbit polyclonal anti-CD44 antibody (1:1000, ab157107, Abcam, Cambridge, MA, USA) was added and incubated for 24 h at 4 °C. Then TBST washes were performed and 100 µl of the secondary antibody (Goat anti-Rabbit conjugated with Alexa Fluor 568, 1:1000; Invitrogen, Carlsbad, CA. USA) was added and incubated for 2 h at RT and in the dark. Subsequently, TBST washes were performed and 200 µl of 4′,6-diamidino-2-phenylindole (DAPI) were added for nuclei contrast. Samples were incubated with DAPI for 30 min at RT, and later the coverslips were mounted with glycerol on slides. Finally, they were analyzed, and high-resolution digital images were obtained by a fluorescence Eclipse 50i microscope (Nikon, Tokyo, Japan) with a NIS-Elements software system (Digital Sight dDS-2Mu).

### 2.5. Analysis of Cells Viability with 3-(4,5-Dimethylthiazol-2-yl)-2,5-Diphenyltetrazolium Bromide (MTT) Colorimetric Assay

For the determination of cell growth and viability, the MTT assay was used, which is based on the reduction of the tetrazolium dye MTT 3-(4,5-dimethylthiazol-2-yl)-2,5-diphenyltetrazolium bromide to its insoluble formazan salt. The amount of formazan produced is proportional to the number of living cells [33]. For this assay, 7500 cells were incubated per well in 100 µL of culture medium in a 96-well plate and the cells were left adherent for 24 h. Subsequently, the medium was removed and 100 µL of culture medium with the treatments (cCNTs, oxCNTs, oxCNT-HA, and oxCNT-HA-CPT) were added. The treatments were sonicated and administered in doses of 0, 5, 10, 30, 50, and 100 µg/mL, then the cells were exposed to the treatments for 12, 24, 48, and 72 h. Finally, 15 µl of the MTT solution (3 mg/mL) were added to each well and incubated for 3.5 h at 37 °C. The medium was then removed and 150 µL of MTT developer (4 mM HCl, 0.01% NP40 in isopropanol) was added. The plates were covered and shaken for 15 min. Finally, the absorbance was read at 590 nm.

### 2.6. Nuclei Contrast Assay with DAPI

For DAPI nuclei contrast assays, 7500 cells per well were incubated with culture medium (100 µL per well) in a 96-well plate. The cells were adherent for 24 h and the medium was subsequently removed; then 100 µL of culture medium were added with the treatments (cCNTs, oxCNTs, oxCNT-HA, and oxCNT-HA-CPT) and incubated for 24 h. After this time, the medium was removed with treatment, and washes were performed with PBS 1X. The cells were then fixed with methanol-acetone in a 1: 1 ratio at 4 °C for 20 min. After fixation washes were performed with PBS 1×, 100 µL of DAPI (1 µL of 1 mg/mL solution) was added and left to incubate at RT and in the dark. After this time, the plates were observed in the fluorescence microscope.

### 2.7. Comparative Assay of the oxCNT-HA-CPT Nanovector vs. CPT Chemotherapy

To determine the efficacy of the nanovector, the previously described MTT assay was used in the cells exposed to treatments with oxCNT–HA–CPT and CPT for 24 h. Also, the nuclei contrast assay with DAPI was performed in these same cells.

## 3. Results

### 3.1. Oxidation and Functionalization Induce Changes in the cCNTs Walls

**Figure 1:**
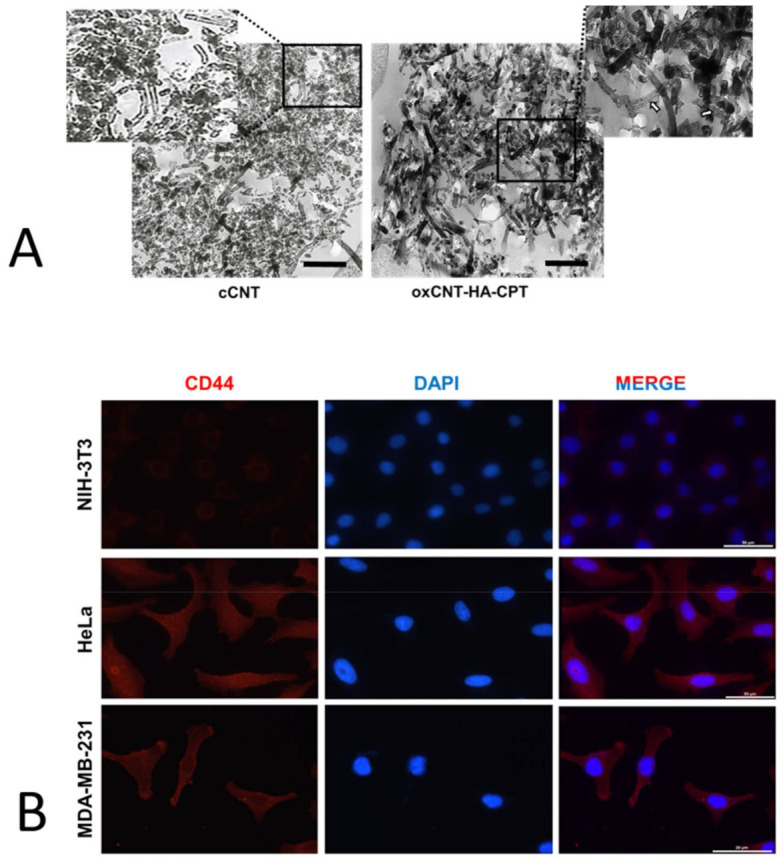
(**A**). Transmission electron micrographs of commercial-grade carbon nanotubes (cCNTs). Notice the disorganization and agglomeration of the nanotubes. In the oxidized and functionalized CNTs loaded with CPT (oxCNTs–HA-CPT), observe the greater scattering, increase in the electrodensity of the walls, and the electrodense spots inside that correspond to the Carboplatin (CPT) (white arrows). Scale = 1.5 µm. (**B**). Determination of the CD44 receptor in NIH-3T3 (non-tumor cells), HeLa (cervical cancer cells), and MDA-MB-231 (breast cancer cells) using the indirect immunofluorescence technique. Note the increased positivity for the CD44 receptor in tumor cells. Scale = 50 µm.

The characterization of the pristine and functionalized nanomaterials cCNTs and oxCNT–HA–CPT, respectively, was performed using Transmission Electron Microscopy (TEM). In pristine cCNTs, the structure is seen as the typical rolled pattern bending on its axis. The walls of cCNTs appear to be electrodense, but nanotubes are found to be thin and empty (Figure 1A, left).

On the other hand, the functionalized oxCNTs–HA-CPT nanovectors showed more electrodense and thicker than pristine cCNTs. Furthermore, subtle dottings can be visualized when zooming in the interior of the oxCNTs–HA–CPT nanovectors; this is attributed to the loading of CPT into the oxCNTs–HA nanostructures (Figure 1A, right).

### 3.2. HeLa and MDA-MB-231 Tumor Cells Show Higher Expression of the CD44 Receptor than Non-Tumor NIH-3T3

Since the overexpression of the HA receptor (CD44) is important to enhance the uptake of the HA-functionalized CNTs through receptor-mediated endocytosis, we analyzed the expression of this membrane receptor in the tumor and non-tumor cells.

For this, the indirect immunofluorescence technique was performed. The first column of micrographs (Figure 1B, left) corresponds to the CD44 receptor protein detection, which can be observed in red color. It is observed that the red color intensity is quite low in NIH-3T3 non-tumor cells, showing a very low expression of the receptor in this cell line. On the other hand, in HeLa and MDA-MB-231 tumor cells this signal is seen with greater intensity, which indicates a greater presence of the CD44 receptor in tumor cells. The second column of micrographs (Figure 1B, center) corresponds to the blue channel, which shows the DAPI staining for cell nuclei. The third column corresponds to the merge of red and blue channels (Figure 1B, right). There, the signals for the CD44 receptor and the cell nuclei can be visualized together. As a result, a more significant presence of the CD44 receptor is observed in tumor cell lines than non-tumor cells, which is a condition of great importance to ensure that nanovectors bio-direct towards specific tumor cells.

### 3.3. The Nanodevice Shows a Greater Cytotoxic Effect vs Tumor Cells and Less against Non-Tumor Cells

Both HeLa and MDA-MB-231 tumor cells and NIH-3T3 non-tumor cell lines were treated with different treatments and doses. The potential cytotoxic effect of the nanovectors was evaluated using light microscopy and analyzing the morphology of the cells, cytoplasm reduction and loss of adherence to the plate are related to cell stress and death respectively. 

**Figure 2:**
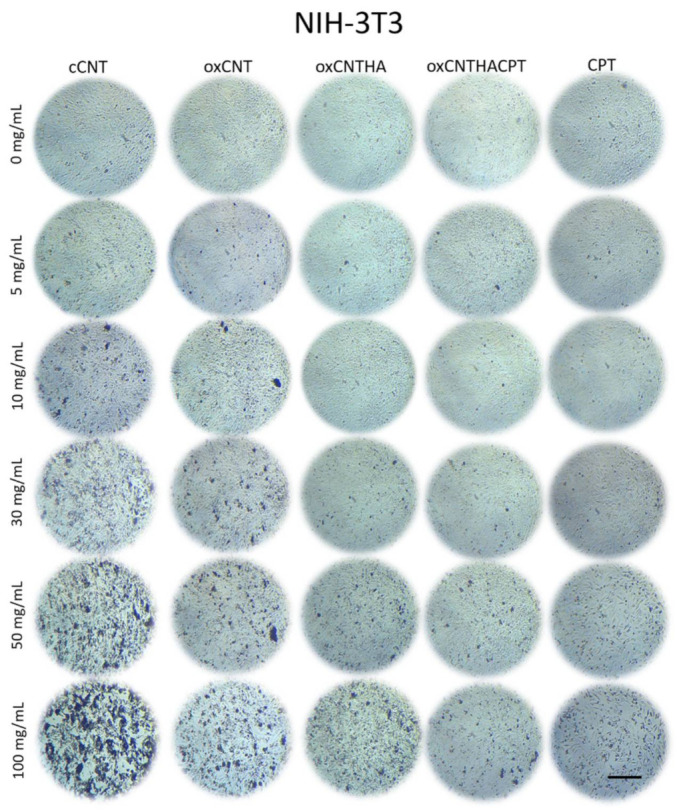
Micrographs of NIH-3T3 exposed for 24 h to 0, 5, 10, 30, 50 and 100 µg/mL. Note that the higher the concentration of the commercial-grade carbon nanotubes (cCNT), the greater the agglomeration of the nanoparticles, and the lower of cell confluence. In the oxidized CNTs (oxCNTs), oxidized and functionalized CNTs (oxCNT–HA), and the oxidized and functionalized CNTs loaded with CPT (oxCNTs–HA-CPT) treatments, the higher the concentration of the treatment the fewer agglomerates, and the cell confluence does not decrease significantly. Interestingly, in the Carboplatin (CPT) treatments, note that the higher the concentration, the lower the cell confluence. The cells detach from the well due to the cytotoxicity of the treatment and change their morphology. Scale = 200 µm.

**Figure 3:**
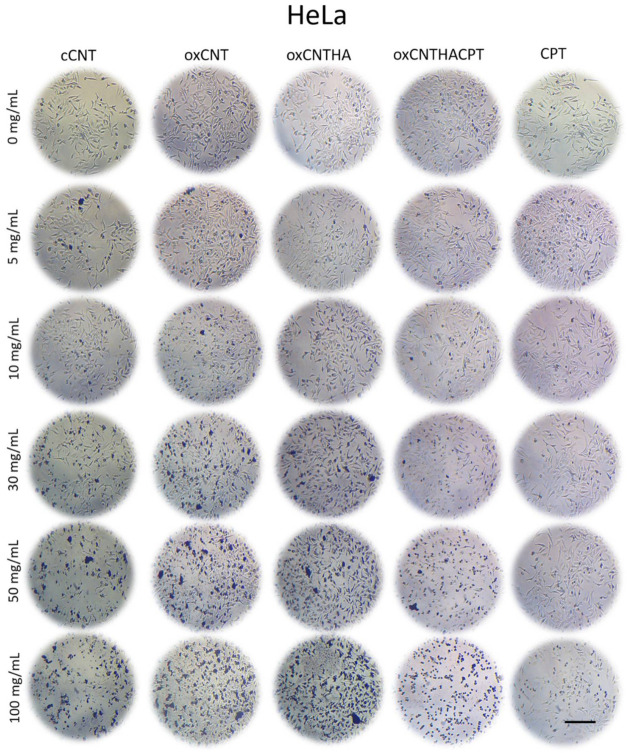
Micrographs of HeLa cells in culture and exposed for 24 h at concentrations of 0, 5, 10, 30, 50, and 100 µg/mL. Note that the higher the concentration of cCNTs, the greater the agglomeration of the nanoparticles, and the lower the cell confluence. On the contrary, in the oxCNTs and oxCNT–HA treatments, it is observed that the higher the concentration, the nanoparticle agglomerates are less, and the cell confluence does not decrease significantly. Interestingly, in the treatments with oxCNT–HA–CPT and CPT, it was observed that the higher the concentration of the treatment, the lower the cell confluence. The cells detach from the well due to the cytotoxicity of the treatment and change their morphology. However, the toxicity of CPT is lower compared to that of oxCNT–HA–CPT. Scale = 200 µm.

The MTT metabolic activity assay was used to determine cell viability. Bright-field photomicrographs for NIH-3T3 (Figure 2) and HeLa cell (Figure 3) cultures are used to compare different treatments with the prepared nanovectors. After 24 h, the cytotoxic effect can be observed as a change in cell morphology followed by a decrease in their adherence to the plaque. In particular, the cytotoxic effect of cCNTs was observed in the two cultures at higher concentrations of nanomaterials; the presence of cCNTs agglomerates was also evident in plaques. In the NIH-3T3 cell culture treated with oxCNTs and oxCNTs–HA, a low cell cytotoxic effect was observed, with a much less noticeable presence of agglomerates. NIH-3T3 treated with the oxCNTs–HA–CPT nanovector resulted in a non-significant cytotoxic effect even though the presence of the antineoplastic CPT.

On the other hand, a cytotoxic effect can be observed in the HeLa cells when the cell culture is treated with cCNTs; however, the treatments using oxCNTs and oxCNTs–HA showed lower cytotoxic effects. This result can be attributed to the low level of agglomeration. However, the oxCNTs–HA–CPT nanovector increases the cytotoxicity effects on HeLa cells, and these effects were dose-dependent. For comparison, CPT is cytotoxic for non-tumor cells at a higher concentration (Figure 2) but, at the same time, the CPT treatment is not cytotoxic to the HeLa tumor cells (Figure 3) even with the highest concentration employed (100 µg/mL).

### 3.4. The oxCNTs-HA-CPT Nanovector Exhibits a Potential Cytotoxic Activity Compared to CPT in Tumor Cells

**Figure 4:**
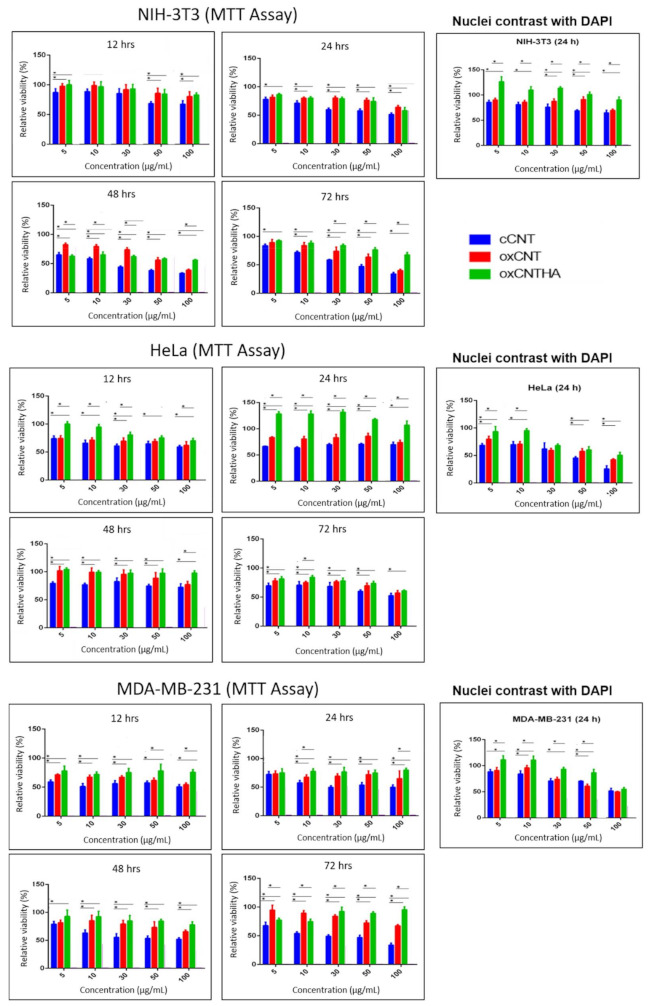
Graphs of the 3-(4,5-dimethylthiazol-2-yl)-2,5-diphenyltetrazolium bromide (MTT) colorimetric, and 4′,6-diamidino-2-phenylindole (DAPI) assays representing viability in NIH-3T3, HeLa, and MDA-MB-231 cell lines after administration of cCNT (blue bar), oxCNT (red bar), and oxCNT-HA (green bar) at doses of 0, 5, 10, 30, 50 and 100 µg/mL and 12, 24, 48, and 72 h. Note that oxidation and functionalization of cCNTs decrease their cytotoxicity. (n = 5). Tukey’s statistical test. *P* < 0.05. The asterisk (*) indicates statistical significance.

**Figure 5:**
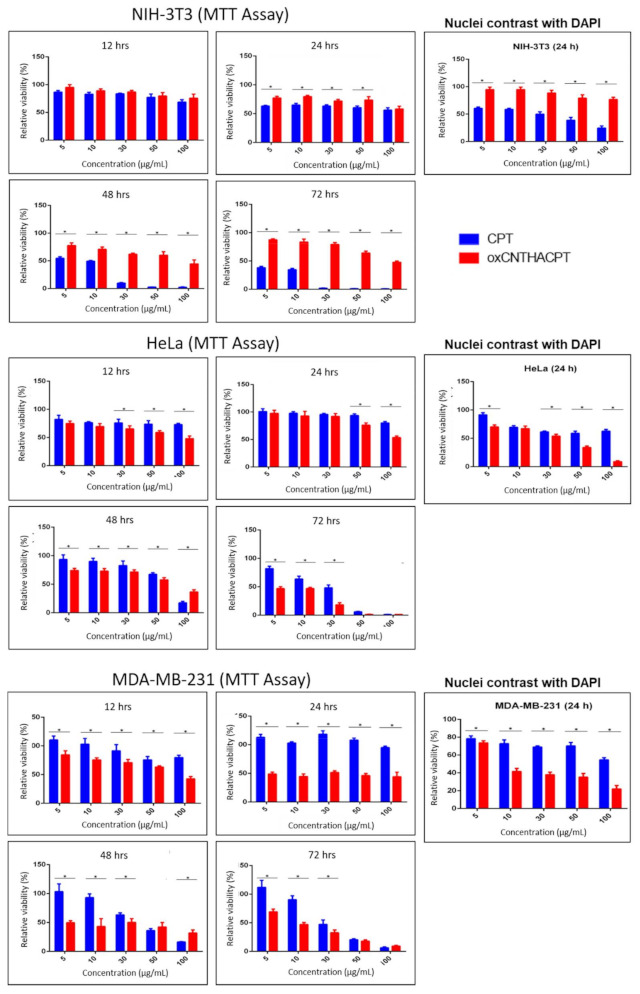
Graphs of the MTT and DAPI assays that represent the viability of the NIH-3T3, HeLa, and MDA-MB-231 cell lines after administration of CPT (blue bar) and oxCNT-HA-CPT (red bar) at doses of 0, 5, 10, 30, 50, and 100 µg/mL and 12, 24, 48 and 72 h of exposure. Note that, after 24 h of exposure, CPT has a greater cytotoxic effect on non-tumor cells, compared to oxCNT-HA-CPT. On the contrary, the oxCNT–HA–CPT nanovector has a greater cytotoxic effect on tumor cells compared to CPT. (n = 5). Statistical test of Student’s T. *P* < 0.05. The * indicates statistical significance.

The efficacy of the oxCNTs–HA–CPT nanovector against tumor cells was evaluated and compared with the CPT treatment. It was observed that both treatments caused similar cytotoxicity at 12 h in the viability tests for NIH-3T3 cells. However, after 24 h, CPT showed more significant cytotoxic activity against non-tumor cells. The cytotoxicity effect of CPT dramatically decreased the cell viability after 48 h, even at low doses of the drug. At the same time, the oxCNTs–HA–CPT treatment did not show a drastic cytotoxic effect in the NIH-3T3 non-tumor cell line (Figure 4 and Figure 5).

HeLa tumor cells exposed to oxCNTs–HA–CPT and CPT treatments resulted in similar cytotoxicity effects at low concentrations doses after 12 h and 24 h. However, oxCNTs–HA–CPT showed a cytotoxic activity after 48 h. In comparison, CPT requires higher doses to achieve a cytotoxic effect similar to the treatment using oxCNTs–HA–CPT (Figure 4 and Figure 5).

The MTT viability tests in the MDA-MB-231 tumor cells exposed to oxCNTs–HA–CPT, and CPT, at different doses and concentrations, were similar to those observed in HeLa cells. The oxCNTs–HA–CPT treatment showed more significant cytotoxic activity than CPT after 12 h, while CPT treatment requires higher doses to achieve a cytotoxic effect similar to the treatment using oxCNTs–HA–CPT (Figure 4 and Figure 5).

The results obtained from the MTT assay were confirmed with DAPI. Graphs of the MTT and DAPI assays representing viability in NIH-3T3, HeLa, and MDA-MB-231 cell lines after administration of cCNTs, oxCNTs, oxCNTs–HA, and oxCNTs–HA–CPT nanovectors at doses of 0, 5, 10, 30, 50, and 100 μg/mL and 12, 24, 48, and 72 h are shown in Figure 4. The treatments in both tumor and non-tumor cell lines were evaluated using similar concentrations at 24 h of exposition. DAPI analysis indicates that the oxCNTs–HA–CPT treatment resulted in lesser toxicity against NIH-3T3 non-tumor cells, while CPT yields more significant cytotoxicity against NIH-3T3. On the contrary, the oxCNTs–HA-CPT treatment for tumor cells showed a more significant cytotoxic effect than CPT (Figure 4 and Figure 5). These results are attributed to the functionalization with HA, which allows nanovectors to be bio-directed towards tumor cells.

In the MTT analysis in MDA-MB-231 cells, similar results to HeLa cells were observed in this cell-line at the same concentrations and exposure times to the treatments (images not shown, Figure 4 and Figure 5).

## 4. Discussion

Nanomedicine is the application of nanotechnology for medical purposes, which includes the of nanomaterials for the diagnosis, monitoring, prevention, and treatment of diseases [8]. CNTs can be excellent vehicles for drug delivery because their high surface area, excellent chemical stability, and rich electronic polyaromatic structure allow conjugation with a wide variety of therapeutic agents [34]. Emerging biomedical applications of CNTs, specifically for therapy, will depend upon whether these nanostructures, either pristine or functionalized, can be proven to be safe beyond doubt. Considering that drug delivery to cells is safer and more effective than conventional therapies [35], several studies have shown that CNTs are excellent carriers because CNTs can penetrate directly into cells and deliver the drug intact and unmetabolized during transport [36]. In the present study, a nanovector prepared from MWCNTs (outside diameter, 30–80 nm) was oxidized and functionalized with HA and filled with CPT, following a previous methodology [29].

Our previous study [29] showed that oxCNTs–HA–CPT have an excellent and specific antitumor effect in vitro, and the cytotoxic effect of the as-prepared nanovectors was demonstrated significantly higher in murine tumor cells TC-1 [29]. The application of such nanovector in an in vivo tumor model serves to know its effects in a broader sense. The human cervical adenocarcinoma (HeLa) and the mammary adenocarcinoma (MDA-MB-231) cell lines were chosen because they have previously been described as over-expressing CD44 [37]. The present study showed that the oxCNTs–HA–CPT nanovector is selective against HeLa and MDA-MB-231 due to the increased binding to the CD44 receptor and subsequent endocytosis. On the contrary, the cytotoxic effect of the nanovector was not meaningful in non-tumor cells. Hence, the prepared nanovector demonstrates a potential antineoplastic effect against murine tumor cells [29] and over human cancer cells in vitro.

The exploration of appropriate cell lines to investigate specific receptors and alternatives for therapeutic approaches can be enhanced by their ability to penetrate biological membranes with relatively low cytotoxicity once the nanovector is ready and administered to the cells. This study showed that the oxidation and functionalization of cCNTs decreased their cytotoxicity, thus producing a safe nanovector for drug delivery. CNTs can be opened, filled, and closed again without losing their stability [38]. Moreover, CNTs can be filled or loaded with a chemotherapeutic agent. Therefore, the toxicity effect is attributed mainly to CPT in a dose-dependent manner. First, the functionalization of the nanovector was compared and monitored along the external sidewalls of CNTs. Micrographs with TEM showed sidewalls thin, with mild electron density, and empty in cCNTs, while the sidewalls are thicker due to oxidation and functionalization processes in the nanovector; such sidewalls appeared to be occupied with CPT. TEM is described as an adequate method of morphological characterization for CNTs because it allows visualizing changes in the electron density of the sidewalls without damaging or modifying the structure [39]. Second, it was necessary to demonstrate whether cell lines overexpress the HA receptor (CD44). The indirect immunofluorescence technique showed the increased positivity for the CD44 receptor in tumor cells. Then, the functionalization of oxCNTs with HA facilitates that nanovectors bio-direct toward tumor cells that over-express this CD44 receptor producing endocytosis [12]. Finally, the efficacy of the nanovector was compared with CPT. It was found that the nanovector was cytotoxic to human tumor cells after 12 h of exposure, while CPT showed a lower cytotoxic effect compared to the nanovector. Higher doses of CPT were required to achieve a nanovector-like cytotoxic effect. Importantly, it was observed that the nanovector did not show a significant cytotoxic effect, while the CPT showed an evident cytotoxic effect in non-tumor cells. These results demonstrated that the nanovector bio-directs towards tumor cells, producing a selective cytotoxic effect on them.

Although conventional therapy against cancer depends on its detection and diagnosis stage, chemotherapy is required when the stage is advanced using platinum compounds. However, the response is highly variable, not consistently favorable, and not without severe adverse effects [40]. Furthermore, this study sought to expand the application and evaluation of novel alternative therapeutics based on nanovectors fabricated with CNTs. As a perspective, it remains to investigate whether these effects are maintained with other human cell lines and in an in vivo analysis, in which it would be expected to find a significant decrease in the adverse effects usually caused by the use of conventional chemotherapy. To evaluate the potential antineoplastic response and their adverse effects of the oxCNTs–HA–CPT nanovector, our group is currently evaluating the application of treatments in other human tumor cell lines and the in vivo evaluation in murine models.

## 5. Conclusions

It was shown that human tumor cell lines overexpress the HA receptor (CD44), which enhances the uptake of the nanovectors through receptor-mediated endocytosis. On the other hand, low expressing CD44 (as NIH–3T3) cells have a lower efficiency to capture and internalize the HA-functionalized CNTs.

Through oxidation and HA–functionalization processes in the nanotubes, it was possible to decrease their cytotoxicity in both human tumor cells (HeLa, and MDA–MB–231) and murine non-tumor cells (NIH–3T3), showing that oxCNTs–HA are safe and biocompatible vectors for CPT delivery. The prepared nanovector produces negligible cytotoxic effects in non-tumor cells than the ones caused by CPT. On the other hand, in human tumor cells, it showed a more significant cytotoxic effect than CPT, which is attributed to the biodirectioning of the nanovector. The oxCNTs–HA–CPT nanovector showed cytotoxic activity against human tumor cells in vitro.

## Data Availability

Data are contained within the article.
